# A New Mesenchymal Stem Cell (MSC) Paradigm: Polarization into a Pro-Inflammatory MSC1 or an Immunosuppressive MSC2 Phenotype

**DOI:** 10.1371/journal.pone.0010088

**Published:** 2010-04-26

**Authors:** Ruth S. Waterman, Suzanne L. Tomchuck, Sarah L. Henkle, Aline M. Betancourt

**Affiliations:** 1 Department of Anesthesiology, Tulane University, New Orleans, Louisiana, United States of America; 2 Department of Microbiology and Immunology, Tulane Cancer Center, Tulane Center for Gene Therapy, Tulane University, New Orleans, Louisiana, United States of America; New York University, United States of America

## Abstract

**Background:**

Our laboratory and others reported that the stimulation of specific Toll-like receptors (TLRs) affects the immune modulating responses of human multipotent mesenchymal stromal cells (hMSCs). Toll-like receptors recognize “danger” signals, and their activation leads to profound cellular and systemic responses that mobilize innate and adaptive host immune cells. The danger signals that trigger TLRs are released following most tissue pathologies. Since danger signals recruit immune cells to sites of injury, we reasoned that hMSCs might be recruited in a similar way. Indeed, we found that hMSCs express several TLRs (e.g., TLR3 and TLR4), and that their migration, invasion, and secretion of immune modulating factors is drastically affected by specific TLR-agonist engagement. In particular, we noted diverse consequences on the hMSCs following stimulation of TLR3 when compared to TLR4 by our low-level, short-term TLR-priming protocol.

**Principal Findings:**

Here we extend our studies on the effect on immune modulation by specific TLR-priming of hMSCs, and based on our findings, propose a new paradigm for hMSCs that takes its cue from the monocyte literature. Specifically, that hMSCs can be polarized by downstream TLR signaling into two homogenously acting phenotypes we classify here as *MSC1* and *MSC2*. This concept came from our observations that TLR4-primed hMSCs, or *MSC1*, mostly elaborate pro-inflammatory mediators, while TLR3-primed hMSCs, or *MSC2*, express mostly immunosuppressive ones. Additionally, allogeneic co-cultures of TLR-primed MSCs with peripheral blood mononuclear cells (PBMCs) predictably lead to suppressed T-lymphocyte activation following *MSC2* co-culture, and permissive T-lymphocyte activation in co-culture with *MSC1*.

**Significance:**

Our study provides an explanation to some of the conflicting reports on the net effect of TLR stimulation and its downstream consequences on the immune modulating properties of stem cells. We further suggest that MSC polarization provides a convenient way to render these heterogeneous preparations of cells more uniform while introducing a new facet to study, as well as provides an important aspect to consider for the improvement of current stem cell-based therapies.

## Introduction

Multipotent mesenchymal stromal cells (formerly known as mesenchymal stem cells, MSCs) are readily separated from other bone marrow-derived cells by their tendency to adhere to plastic. MSCs differentiate into osteoblasts, chondrocytes, and adipocytes under appropriate culture conditions [1]–[4]. Further, they offer the advantage that they are easily expanded and stored *ex vivo* and are considered to be “immunoprivileged.” Thus, once harvested they can safely be infused into either autologous or allogenous hosts owing to their lack of host immune reactivity [2]. These cells home to damaged tissues and contribute to their repair by secretion of cytokines, chemokines, and extracellular matrix proteins [3], [5]. As a result of these qualities, MSCs are very attractive candidates in stem cell-based strategies for tissue repair and gene therapy. Numerous investigators have now demonstrated the successful recruitment and multi-organ engraftment capability of infused MSCs in various animal models and human clinical trials [6]–[10]. However, the precise molecular mechanisms governing stem cell fate, mobilization, and recruitment to the sites of engraftment are not fully understood. Additionally, even though there is a clear clinical benefit observed when MSCs have been used in cell-based therapy, few infused cells (0.1–1%) have been found at the target site [2], [11], [12]. This observation has prompted investigators to suggest that the benefit observed is due to local immune modulation by these cells rather than by differentiation or replacement of the damaged target tissue by the infused stem cells [9]–[11].

Our laboratory and others established a connection between the stimulation of specific Toll-like receptors (TLRs) and the immune modulating responses of human multipotent mesenchymal stromal cells (hMSCs) [13]–[15]. Toll-like receptors recognize “danger” signals and their activation leads to profound cellular and systemic responses that mobilize innate and adaptive host immune cells [16]–[20]. The TLRs consist of a large family of evolutionarily conserved receptors (e.g.-TLR1-9). The danger signals that trigger TLRs are released following most tissue pathologies. Exogenous danger signals typically released after microbial infections are exemplified by endotoxin or lipopolysaccharide (LPS) sheddings. Endogenous danger signals spilled into the circulation from aberrant or wounded cells are characterized by intracellular components like heat shock proteins or RNA. Typically, these shed danger signals activate TLRs on sentinel innate immune cells (e.g.-dendritic cells), and start an appropriate host response that re-establishes homeostasis [16]–[19]. Since danger signals recruit immune cells to sites of injury we reasoned that hMSCs might employ the same mechanisms to find the tissues in need of their reparative mission. Surprisingly, we found that not only did hMSCs express several TLRs but also that their capability to migrate, invade, and secrete immune modulating factors was drastically affected by specific TLR-agonist engagement [15]. In particular, we observed that TLR3 stimulation leads to the secretion of factors with mostly immune suppressive properties, while stimulation of TLR4 with LPS resulted in the secretion of more pro-inflammatory factors.

Other investigations have evaluated the effects of TLR engagement on the typical stromal stem cell properties of tri-lineage differentiation (chondrogenic, osteogenic, adipogenic) and proliferation. For instance, Hwa Cho *et al.* described a role for TLRs in proliferation and differentiation of human adipose-derived stem cells (hADSCs) [13]. In another report, murine MSC (muMSCs) were found to express TLRs that upon activation affected their proliferation and differentiation [14]. However, in contrast to hMSCs, they suggested that activation of TLR2 inhibits both differentiation and migration of muMSCs while promoting their proliferation. Liotta *et al.* found no effect of TLR activation on adipogenic, osteogenic, or chondrogenic differentiation in hMSCs [21]. Further, in contrast to our study, their report suggested equivalent roles for TLR3 and TLR4 engagement in hMSC immune modulation. Recently, Lombardo *et al.* reported that TLR3 and TLR4 engagement within hADSCs increased osteogenic differentiation but had no effect on their adipogenic differentiation or proliferation [22]. They also concluded that TLR2, TLR3, and TLR4 ligation does not affect hADSCs ability to suppress lymphocyte activation, in contrast to the Liotta *et al.* report.

The recently described immune modulating properties of these cells appear to be rather complex. For instance, immune modulation by hMSCs is attributed to not only secretion of soluble factors but is also dependent on MSC-to-immune cell contact [23]. MSCs express low levels of human leukocyte antigen (HLA) major histocompatibility complex (MHC) class I, do not express co-stimulatory molecules (B7-1, -2, CD40, or CD40L), and can be induced to express MHC class II and Fas ligand explaining why they do not activate alloreactive T cells. MSCs inhibit dendritic cell (DC) maturation, B and T cell proliferation and differentiation, as well as attenuate natural killer (NK) cell killing, and also support suppressive T regulatory cells (Tregs) [3], [23]–[26]. Several factors have been associated with these immune modulating properties of MSCs, including transforming growth factor beta (TGFβ), hepatocyte growth factor (HGF), HLA-G, prostaglandin (PGE_2_), IL-10, indoleamine 2,3-dioxygenase (IDO), and interferon-gamma (IFNγ) [3], [27]–[33]. More recently a role for the notch family member Jagged1 in immune modulation was specifically attributed to downstream TLR signaling of MSCs [21].

Whis this in mind, an explanation for the contrasting and complex immune modulating effects reported thus far for TLR activation in most stem cells may lie in reinterpretation of all of the data taking into account the fact that most of the current stem cell preparations yield heterogeneous pools of cells, as well as acknowledging that TLRs expressed on different cell types and from different species (mouse and man) do not always have the same responses [34], [35]. Other important contributing factors that may account for the conflicting reports in the TLR responses of stem cells are the concentration and length of incubation with the specific TLR agonist, along with careful attention and safeguarding to common LPS (TLR4 agonist) contamination in the laboratory.

Here, we extended our studies on TLRs and immune modulation by hMSCs to provide some support for these concepts and build on our initial observations that low-level, short-term stimulation with specific TLR3 and TLR4 agonists (or TLR-priming) within hMSCs mediates distinct immune modulating responses. It is established that stimulation of monocytes with known cytokines or agonists to their TLRs, like interferon-γ- and endotoxin (LPS, TLR4-agonist), polarizes them into a classical M1 phenotype that participate in early pro-inflammatory responses, while IL-4 treatment of monocytes yields the alternate M2 phenotype that is associated with later anti-inflammatory resolution responses [36]. We introduce a new aspect of hMSC biology implied by this work, that suggests that hMSCs, like monocytes, are polarized by downstream TLR signaling into two homogenously acting phenotypes we classify here as MSC1 and MSC2, following the monocyte nomenclature. We suggest that hMSCs respond in a manner analogous to monocytes following specific TLR priming that ultimately will help make MSC preparations more uniform, and will be important to study and consider in future improved designs of stem cell-based therapies [36]–[38].

## Results

### Cytokine and chemokine secretion patterns following TLR3 or TLR4 activation of hMSCs are consistent with divergent immune modulating effects by these agonists

**Figure 1:**
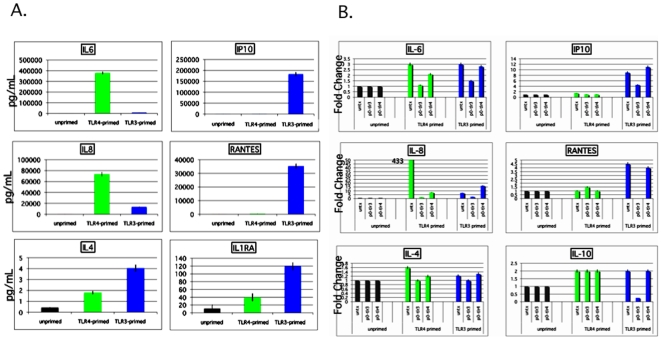
MSC1 differ from MSC2 in their secretion of immune modulators. **A. The data show increased expression of known immune suppressive factors by TLR3-primed hMSCs (MSC2) but not by TLR4-primed hMSCs (MSC1).** *Methods:* MSCs were pre-treated for 1 hr with TLR agonists (LPS for MSC1 or poly(I∶C) for MSC2), washed and cultured for an additional 48 hr prior to harvesting the spent medium and analysis with Bio-Plex Cytokine Assays (Human Group I & II; Bio-Rad, Hercules, CA) following the manufacturer's instructions. Data are presented by the quantitative comparative CT (threshold value) method [46]. Error bars indicate SEM. Data are representative of triplicate measurements with 5 MSC donors. **B. The data implicate direct TLR3 induction of IP10 (CCL10) and RANTES (CCL5) secretion.** *Methods:* hMSCs were transfected with pZERO-hTLR3 and pZERO-hTLR4 (Invivogen), using 250 ng plasmid/1×10^6^ cells (nucleofector). Cells from each transfection were left untreated or stimulated with TLR3 and TLR4 agonists for 1 hr washed and incubated for 48 hr. Conditioned medium was harvested and analyzed as in **A**. Transfection efficiency was determined by these methods to be 30–35%. Data are representative of triplicate measurements with 3 MSC donors.

In this study, we set out to extend our previous observations of the effect that TLR signaling has on the immune modulating property of hMSCs, as well as to potentially provide an explanation for the contrasting reports in this field [15]. In the experiments included here, we typically used a TLR-priming protocol that is defined as the incubation with LPS (10 ng/mL) or poly(I∶C) (1 µg/mL) added as the hMSCs agonists for TLR4 and TLR3, respectively, for no longer than 1 hr prior to washing and further 24–48 hr incubation in growth medium [15]. The short incubation time (<1 hr) and minimal TLR agonist concentrations used here are postulated to mimic the gradient of danger signals endogenous MSCs encounter and respond to at a distance from the site of injury. The conditioned medium was collected and analyzed with Bio-Plex Cytokine Assays (Human Group I & II). Consistent with our published results, TLR3 stimulation in hMSCs led to elevated secretion of certain immune modulating factors different from those elaborated by TLR4 activation in hMSCs (Fig. 1A)[3]. To provide further support for the specific effects by each of these receptors, hMSCs were transfected with dominant negative plasmids for each of the TLR-receptors, and the factors secreted were once again measured by BioPlex assay. This strategy corroborated the TLR3-driven effect on hMSC secretion of CCL10 (IP-10), CCL5 (RANTES), and to a lesser degree IL4 and IL10. It appeared that TLR4 signaling is upstream of IL6 and IL8 as shown in Fig. 1B.

### The amount of time of TLR agonist exposure affects migration and invasion capabilities of the treated hMSCs

**Figure 2:**
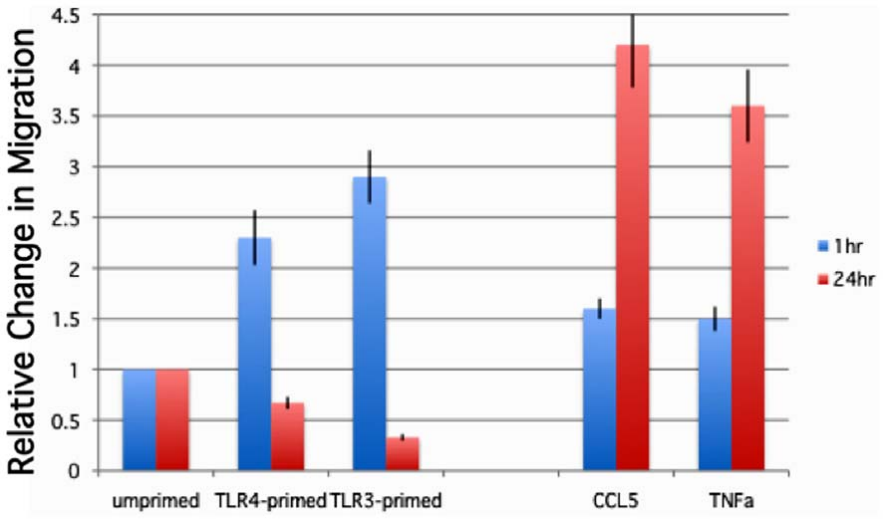
Short-term TLR stimulation promotes the migration of hMSCs. **Data show that short-term TLR-priming stimulates migration. By contrast, 24 hr incubation is needed for enhanced migration by CCL5 (RANTES) and TNFα treatment.** *Methods:* hMSCs migration was examined by transwell migration assay after pre-incubation with TLR-ligands, CCL5 (150 ng/mL), or TNFα (1 ng/mL) for either 1 or 24 hr prior to loading on Matrigel-coated inserts. After overnight incubation, migration towards the serum chemoattractant was visualized and recorded by fluorescence microscopy. Migration was quantified from the obtained micrographs by counting the number of fluorescently-labeled cells remaining after removal of non-migrating cells in triplicate wells. Bar graph of the obtained results normalized to unprimed cells. Error bars indicate SEM. (n = 6).

Apart from the distinct effects of TLR3 and TLR4 activation on cytokine/chemokine secretion we also initially reported that TLR activation promoted hMSC migration, while Pevsner-Fischer *et al.* reported that in murine MSCs TLR activation inhibited the migration of these cells [14]. The hMSC migration assays were performed again with varying incubation times and stimulants to gain a better understanding of this process within hMSCs, and taking into consideration a related report stating that CCL5 (RANTES) driven hMSC migration was highly induced by pre-treatment of the hMSCs with TNFα [39]. Thus, the migration by the TLR-primed hMSCs was analyzed following initial exposure to LPS (TLR4 ligand), poly(I∶C) (TLR3 ligand), CCL5, or TNFα for an hour or 24 hr prior to loading the cells on the top chamber for the transwell migration assays (Fig. 2)[15]. Stimulation for 1 hr of TLR3 and TLR4 within hMSCs promoted migration and invasion towards 16.5% serum containing medium when compared to untreated samples, as previously reported [15]. However, 24 hr incubation with these ligands suppressed migration and invasion of the treated hMSCs. By contrast, this longer incubation time was essential for CCL5 and TNFα driven migration and invasion by the hMSCs. Inhibition of the expression of TLR3 and TLR4 receptors by nucleofection with knockdown plasmids diminished migration by >50% in unprimed hMSCs, consistent with our previous report [15]. However, LPS or poly(I∶C) treatment of the transfected cells resulted in greater migration when compared with unstimulated controls (data not shown). We speculate that the stress of nucleofection and/or the endogeneous inhibition of the TLR receptors may derepress a TLR-associated inhibitor of migration—and thus enhance rather than suppress the migration of transfected hMSCs as we expected. It appears that migration and invasion mechanisms driven by TLRs within hMSCs are more complex than originally appreciated.

### Varying effects of TLR3 and TLR4 stimulation on hMSCs adipogenic and osteogenic differentiation potential

**Figure 3:**
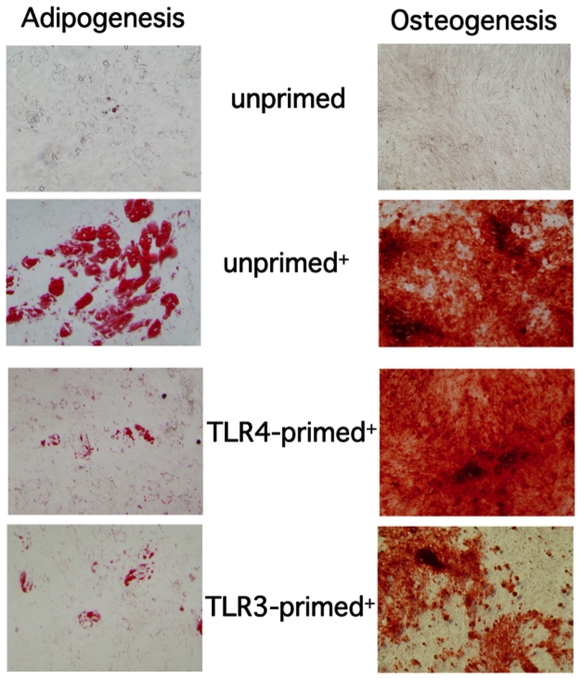
TLR3 activation inhibits bone and fat differentiation. **TLR4 activation promotes bone differentiation and inhibits fat differentiation in hMSCs.** *Methods*: The hMSCs were induced (+) to differentiate in the presence of TLR3 and TLR4 ligands throughout the four-week incubation period prior to staining for differentiation markers by established methods. Untreated hMSCs (untx) were either induced (+) or not and served as assay controls. (n>3)

Next, given the reported differences on the effect that TLR3 and TLR4 activation have on the tri-lineage (cartilage, bone, fat) differentiation capabilities of hMSCs, we also measured these using our particular methods of TLR activation with reduced amounts of TLR ligand when compared to most other studies [13], [14], [22]. The hMSCs were simultaneously induced to differentiate in the constant presence of TLR3 (1 µg/mL poly(I∶C)) and TLR4 agonists (10 ng/mL LPS) maintained for the duration of the differentiation assays in the inductive medium. By this method, we noted an inhibition of all bone, fat, or cartilage (not shown) programs after TLR3 activation of hMSCs (Fig. 3). Simultaneous TLR4 activation of hMSCs inhibited adipogenesis, stimulated osteogenesis, and did not affect chondrogenesis (not shown).

### hMSCs deposit more fibronectin following TLR3 activation, and more collagen when TLR4 is stimulated

**Figure 4:**
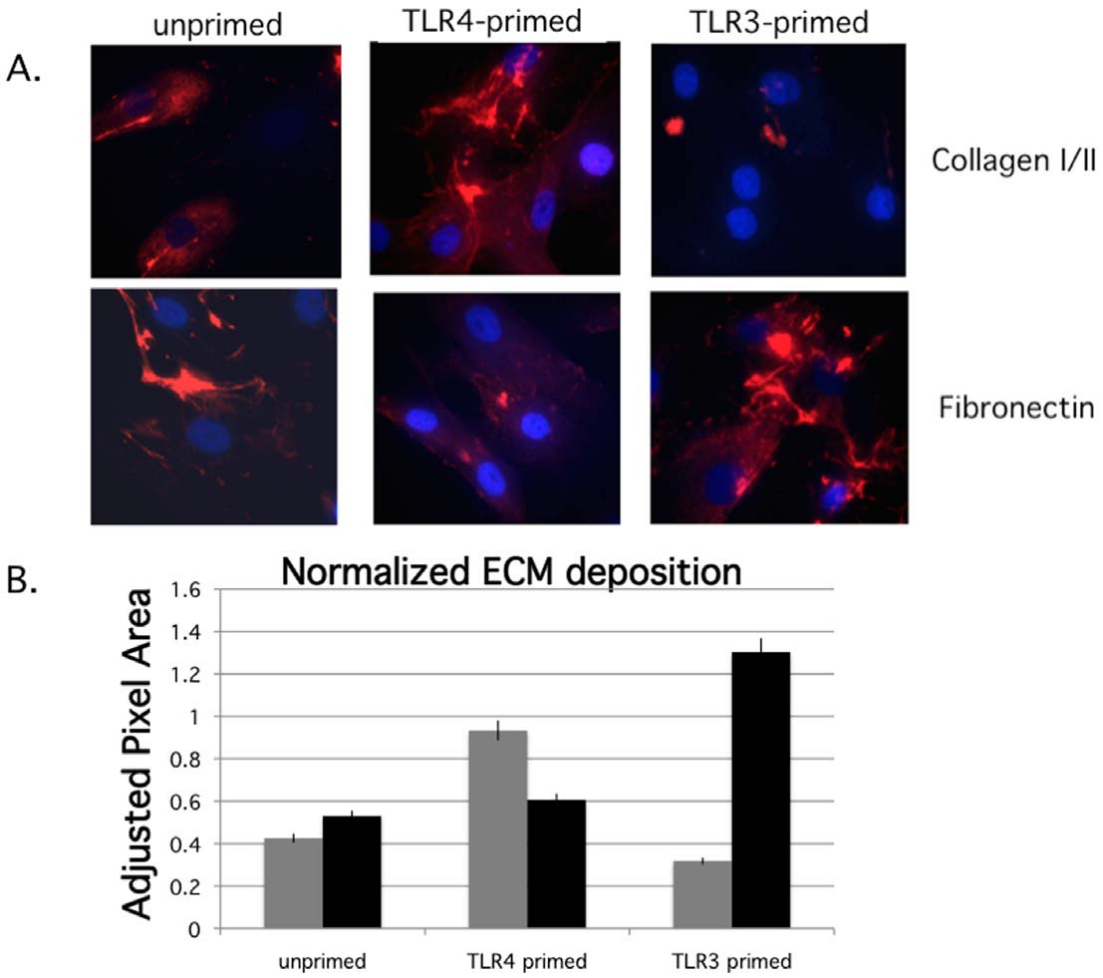
TLR3-primed hMSCs deposit more fibronectin, while TLR4-primed hMSCs deposit more collagen. **A. Data demonstrate that TLR4-primed cells deposit twice as much collagen I/II and half as much fibronectin as TLR3-primed cells. B. Densitometric analysis of micrographs in A. left bars (grey) are collagen I/II and right bars (black) are fibronectin results normalized to background absorbance.** *Methods:* hMSCs were grown on chamber slides to 70% confluence pre-treated for 1 h with ligands: 1 mM poly(I∶C) (TLR3) or 10 ng/mL LPS (TLR 4) and incubated further for 24 hr prior to fixation. ECM antibody staining was performed following fixation and membrane permeabilization of the TLR-primed or unprimed hMSCs seeded on chamber slides (antibodies from Chemicon International, CA, hu fibronectin MAB1926 and collagen I/II MAB3391). As a control, the primary antibody was omitted from staining procedure (data not shown, n>6). Densitometric analysis of the micrographs was performed with ImageJ software.

Since we had thus far seen distinct effects of TLR activation within hMSCs and their secretion of cytokines/chemokines and differentiation, it was of interest to study whether these different effects extended to another established classical role of hMSCs: extracellular matrix deposition. The hMSCs were grown on chamber slides to 70% confluence primed for 1 hr with TLR3- and TLR4-agonists as before, washed and incubated further for 24 hr prior to fixation. ECM antibody staining was performed following fixation and membrane permeabilization of the TLR-primed hMSCs seeded on chamber slides (Fig. 4). As a control, the primary antibody was omitted from staining procedure (data not shown). Densitometric analysis revealed that TLR3 stimulation of hMSCs resulted in diminished collagen I/II deposition when compared to unprimed or TLR4 stimulated hMSCs. This treatment also resulted in greater than two-fold fibronectin deposition when compared to unprimed or TLR4 stimulated hMSCs (Fig. 4B). Interestingly, integrin-linked kinase (ILK) and von Hippel-Lindau protein (VHL), which are also associated with ECM deposition mechanisms, are also differentially expressed after TLR-stimulation. TLR3-stimulation of hMSCs increased the expression of both ILK and VHL, whereas TLR4-stimulation dampened their expression (data not shown).

### Transforming growth factor β (TGF)1,3 secretion is repressed by TLR3 but not TLR4 stimulation of hMSCs

**Figure 5:**
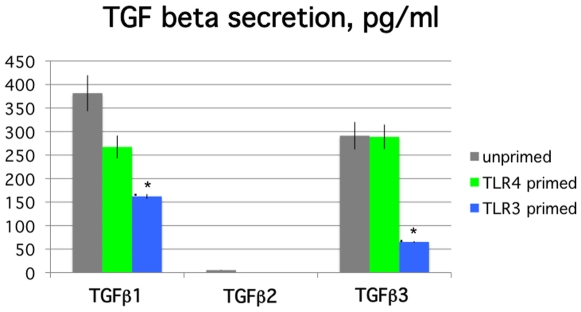
Transforming growth factorβ (TGFβ1 and 3) expression is diminished in TLR3-primed MSCs compared with measured levels for TLR4-primed and unprimed MSCs. **TGFβ 2 levels are small but are further repressed by both treatments.** *Methods:* MSCs were pre-treated for 1 hr with TLR4 agonist (LPS for MSC1) or TLR3 agonist (poly(I∶C) for MSC2), washed, and cultured for an additional 48 hr prior to harvesting the spent medium for TGF**β** detection. TGF**β** 1, 2 & 3 were detected by luminex immunoassay (Luminex® Bead immunoassay Kit, LINCOplex from Millipore). Data are representative of triplicate measurements with 6 hMSC donors. Error bars indicate SEM. *p<0.005 comparison to unprimed MSCs.

TGFβ secretion by hMSCs was measured from the conditioned medium after TLR3 and TLR4 priming, as before (Fig. 5). We were interested in this family of factors, since TGFβ is known to mediate elevated collagen deposition as supported by our TLR4-priming results above, and it is also a known immune modulating factor [40], [41]. TGFβ1, 2, and 3 were measured from the spent culture medium by luminex immunoassay as per manufacturer's recommendations (LINCOplex from Millipore). The TLR-primed hMSCs were washed and cultured for an additional 48 hr prior to harvesting the spent medium for TGFβ detection. TLR3 activation of hMSCs considerably reduced (>65–80%) secretion of TGFβ1 and 3. The levels measured for TGFβ2 secretion were small for all samples (5 pg/mL), and were reduced by both treatments (<1 pg/mL, data not shown). As expected, TLR4 stimulation of hMSCs led to little or no change over the untreated samples for this parameter (data not shown).

### The downstream TGFβ effectors SMAD3 and SMAD7 are differentially expressed by TLR3 and TLR4 priming of hMSCs

**Figure 6:**
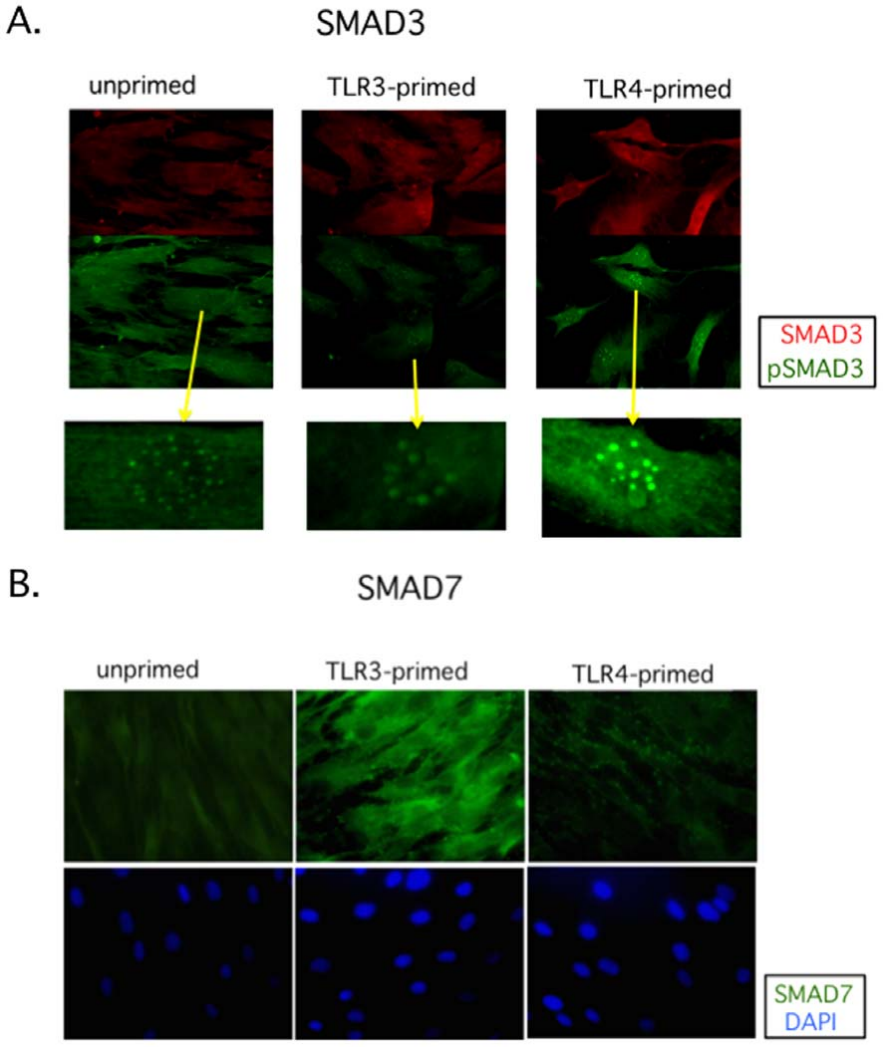
SMAD3 expression and activation (phosphoSMAD3, pSMAD3), as well as SMAD7 expression in hMSCs. **A. Data show that SMAD3 is activated in TLR4-primed (increased nuclear pSMAD3) but not TLR3-primed hMSCs. Yellow arrows point to corresponding magnified cell nuclei. B. SMAD7 expression is induced after TLR3 but not TLR4 stimulation of hMSCs.** *Methods:* hMSCs were grown on chamber slides to 70% confluence, TLR-primed as before, and incubated further for 24 hr prior to fixation. SMAD3, SMAD7, and phosphoSMAD3 antibody staining was performed as indicated in Methods. Representative micrographs of 5 tested hMSC donors.

The downstream TGFβ effectors SMAD3 and SMAD7 that might support the TGFβ results presented above were measured after TLR stimulation of hMSCs. The hMSCs were grown on chamber slides to 70% confluence, pre-treated for 1 hr with TLR3 and TLR4 agonists, washed, and incubated further for 24 hr prior to fixation. Fluorescently labeled SMAD3, phospho-SMAD3 (activated SMAD3), and SMAD7 antibodies were incubated with the TLR-primed hMSCs as indicated (Fig. 6A & B). As a control, the primary antibody was omitted from staining procedure (data not shown). Densitometric analysis revealed that TLR3 stimulation of hMSCs resulted in elevated SMAD7 expression, and diminished and diffused nuclear phospho-SMAD3 and SMAD3, whereas TLR4 stimulation led to increased focused nuclear phosphoSMAD3 expression and reduced SMAD7 expression when compared with untreated hMSCs. Flow cytometry analyses of these markers supported these observations (data not shown).

### Distinct effects are found by TLR3 and TLR4 stimulation in the hMSCs on the expression of Jagged 1 and 2

**Figure 7:**
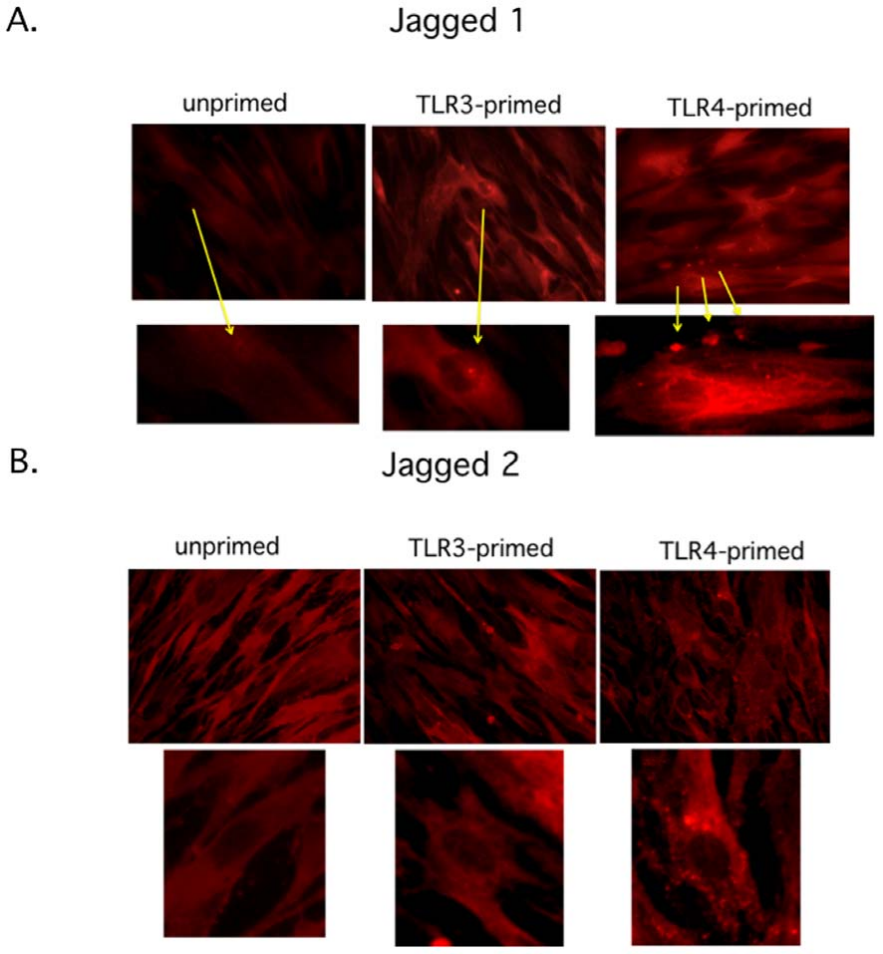
Jagged 1 and Jagged 2 expression in hMSCs. **A. Data show that Jagged 1 expression is elevated, perinuclear, and focused on edges in TLR4-primed but not TLR3-primed hMSCs. Yellow arrows point to corresponding magnified cell nuclei. B. Jagged 2 expression is diffuse in TLR3-primed hMSCS, increased, and perinuclear and endosomal after TLR4 stimulation of hMSCs.** *Methods:* hMSCs were grown on chamber slides to 70% confluence TLR-primed as before and incubated further for 24 hr prior to fixation. Jagged 1 and Jagged 2 antibody staining was performed as indicated in Methods. Representative micrographs of 5 tested hMSC donors.

We next measured Jagged 1 and 2 expression in TLR stimulated hMSCs since these proteins have been linked to some of the controversial reports on immunomodulation following TLR activation of MSCs, and are also known to correlate with TGFβ signaling [21], [42], [43]. The hMSCs were grown on chamber slides to 70% confluence, pre-treated for 1 hr with TLR3 and TLR4 agonists, washed, and incubated further for 24 hr prior to fixation. Fluorescently labeled Jagged 1 and Jagged 2 antibodies were incubated with the TLR-primed hMSCs, as indicated (Fig. 7). As a control, the primary antibody was omitted from staining procedure (data not shown). Jagged 1 and Jagged 2 expression was diffuse in unprimed hMSCs. TLR3 stimulation of hMSCs resulted in reduced and perinuclear Jagged 1 expression, and unremarkable Jagged 2 expression. TLR4 stimulation led to increased Jagged 1 expression that was found both perinuclear and in foci along cell edges. Jagged 2 expression for these cells had a characteristic endosomal distribution. Flow cytometry analyses of these markers supported some of these observations as shown below.

### Indoleamine 2,3-dioxygenase (IDO) and prostaglandin E2 (PGE_2_), other known mediators of hMSC immune modulation, are also differently affected by TLR3 and TLR4 priming

**Figure 8:**
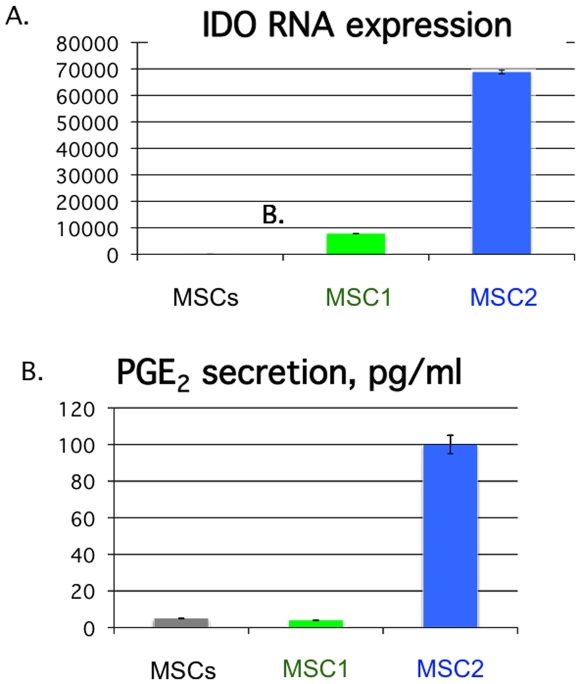
MSC1 differ from MSC2 in their expression of inflammatory mediators. **The data show increased expression of known immune suppressive effectors like indoleamine 2,3-dioxygenase (IDO) and prostaglandin E2 (PGE_2_) by TLR3-primed but not TLR4-primed hMSCs.** *Methods:* MSCs were pre-treated for 1 hr with TLR agonists (LPS for MSC1 or poly(I∶C) for MSC2), washed, and cultured for an additional 48 hr prior to harvesting the spent medium for PGE_2_ detection. PGE_2_ was measured with commercially available ELISA assays (Cayman Chemical, MA). For IDO measurement, MSCs were primed as described, incubated another 6 hr prior to harvesting the RNA and real time PCR assay. Data are presented by the quantitative comparative CT (threshold value) method [56]. Error bars indicate SEM. *n*>3 with at least 4 different hMSC donors.

To lend further support for the observed dichotomy of hMSCs immune modulation downstream from TLR3 and TLR4 stimulation, we also measured other known potentiators of hMSCs immune modulation, IDO and PGE_2_, following the TLR3-and TLR4 priming protocol [26], [44]. IDO was measured by real-time PCR analysis of RNA extracted from TLR-primed hMSCs incubated further for 6 hr prior to RNA harvest. Data are presented by the quantitative comparative CT (threshold value) method (Fig. 8A)[45]. PGE_2_ was measured from the spent culture medium after 1 hr TLR-agonist pretreatment, wash, and 48 hr of subsequent culture by commercially available ELISA assays (Fig. 8B). Consistent with the previous results, it appeared that these immunosuppressive indicators are elevated following TLR3 (poly(I∶C)) stimulation, and, in contrast, mostly unchanged by TLR4 (LPS) activation of the hMSCs.

Arginase and HLA-G expression are other relevant immune modulating effectors that were tested after TLR3 and TLR4 stimulation of hMSCs. These unfortunately gave inconclusive results with the various methods attempted (data not shown).

### Allogeneic co-culture of hMSCs and hPBMCs leads to T cell activation only with TLR4 primed hMSCs but not unprimed or TLR3 primed hMSCs

The immunosuppressive role of heterogeneous MSCs was originally described from allogeneic co-cultures of MSCs with PBMCs or isolated naïve T-cell preparations [3], [31]. The addition of unprimed MSC pools to alloreactive T-cells prevents their activation and/or proliferation. Additionally, MSCs infused into allogeneic hosts do not elicit host immune reactivity. This is largely due to the fact that the unprimed MSCs express low levels of human leukocyte antigen (HLA) major histocompatibility complex (MHC) class I, do not express co-stimulatory molecules (B7.1/CD80, B7.2/CD86, CD40, or CD40L), and can be induced to express MHC class II and Fas ligand only upon interferon treatment [3], [32].

**Figure 9:**
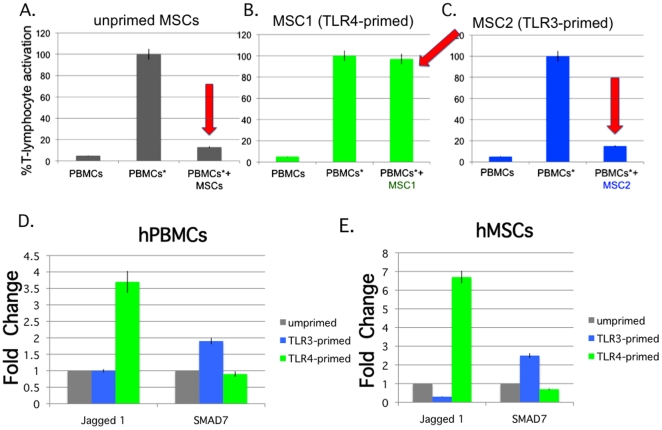
MSC1 support PBMC (T cell) activation, while unprimed MSCs and MSC2 suppress it. The data show differences (red arrows) in T cell activation when allogeneic PBMCs are stimulated (PBMCs*), and co-cultured with untreated MSCs (A), MSC1 (B) or MSC2 (C). 9D and 9E. Expression of Jagged 1 and SMAD7 in MLMR co-culture assays. There is elevated Jagged 1 expression in MLMR assays with MSC1 (TLR4-primed), when compared to MSC2 (TLR3-primed), and unprimed assay cultures. By contrast, there is elevated SMAD7 expression in MSC2, when compared to MSC1, and unprimed assay cultures. 9D. Expression of Jagged 1 and SMAD7 among the CD45+ non-adherent ***hPBMCs*** **collected at the end of the MLMR experiments. 9E. Expression of Jagged 1 and SMAD7 among the CD90+ adherent** ***hMSCs*** **collected at the end of the MLMR experiments.** *Methods:* T cells among the peripheral blood mononuclear cells (PBMCs) were activated with 1 µg of CD3/CD28 antibody beads, prior to labeling with fluorescence label (CFSE), to monitor their activation or cell division, and loaded at a 10∶1 ratio over the hMSCs. The hMSCs were untreated, primed for 1 hr with TLR-4 (MSC1), or TLR3 (MSC2) agonist, washed in medium, and loaded with the PBMCs. After greater than 72 hr of co-culture, the CFSE-labeled PBMCs were harvested from the adherent MSCs, stained with propidium iodide to gate for live cells, and measured by flow cytometry. Unstained cells and PBMCs not activated with antibodies served as controls in the assay. Data are expressed as change from the % T cell activation obtained for CD3/CD28 antibody –activated PBMCs not co-cultured with MSCs = 100. Error bars indicate SEM. Data are averages of triplicate determinations with 5 MSC donors and 2 PBMC donors.

T-lymphocytes among human peripheral blood mononuclear cells (hPBMCs, 10^6^ from at least 5 unrelated donors, labeled), in the presence or absence of the isolated TLR-primed MSCs or unprimed MSCs, were resuspended and stimulated with 1 µg of CD3/CD28 antibody beads. After 72 hr, the cells were stained with CD8- or CD4-antibody, and CFSE-label dilution of the CD8+ cells was assessed by flow cytometry analysis. Data are expressed as percent activation or change from the % T-lymphocyte activation obtained for the activated hPBMCs not co-cultured with hMSCs (Fig. 9)[3]. As previously reported, incubation of unprimed hMSCs with hPBMCs considerably reduced their activation to >90% [3]. However, TLR4 stimulation inhibited this immune dampening effect by the hMSCs (back to almost 100% activation), while TLR3 supported the immune suppression (>90%). Based on these observations, we suggest that TLR-priming effectively polarizes the hMSCs towards two distinct phenotypes. TLR4-priming of hMSCs results in a pro-inflammatory signature we refer to here as *MSC1*; whereas, TLR3-priming supports an immune suppressive one we term *MSC2*. TLR4 activation of hMSCs also consistently resulted in twice as many non-adherent cells recovered at the end of the experiment when compared to the cells recovered from un-activated PBMCs, unprimed hMSC, or TLR3-primed hMSC (Table 1).

**Table 1:** Cell counts after hMSC-PBMC co-cultures.

MSCs	Primed TLR	Leukocyte activation	PBMCs, d1	PBMCs, d2
-	-	+	50,000+/−1784	30,000+/−1774
MSCs, d1	-	+	40,000+/−1352	30,000+/−1980
MSC1, d1	TLR4	+	*70,000*+/−3234	*80,000*+/−5976
MSC2, d1	TLR3	+	35,000+/−1122	33,000+/−1444
MSCs, d2	-	+	50,000+/−2354	40,000+/−1730
MSC1, d2	TLR4	+	*70,000*+/−4376	*80,000*+/−6118
MSC2, d2	TLR3	+	30,000+/−2974	32,000+/−1750

Allogeneic co-culture assays reveal that TLR4 priming of hMSCs (MSC1) promotes T-cell proliferation, while unprimed hMSCs and TLR3 primed hMSCs (MSC2) suppress it. *Methods:* T cells among the peripheral blood mononuclear cells (PBMCs) were activated with 1 µg of CD3/CD28 antibody beads prior to labeling with fluorescence label (CFSE) to monitor their activation or cell division and loaded at a 10∶1 ratio over the MSCs for 72 hr. For cell counts, an aliquot of the 72 hr spent medium was removed prior to flow cytometry for Trypan Blue staining and counting as standard. Data are representative of four independent experiments and are expressed as mean cell counts +/−SEM of 4 replicate counts on a hemocytometer after trypan blue staining. Total of 5 MSC donors and 5 PBMC donors were used in the assay. Two representative donors (d1, d2) are shown.

The expression of various immune modulating factors was measured from the spent co-culture medium at the end of the experiment with BioPlex assays, as before [46]. The expression of CCL5 and CCL10 followed the same patterns as above. Increased secretion for these was observed in co-cultures with TLR3-primed hMSCs when compared to unprimed or TLR4-primed cultures. By contrast, IL6 and IL8 secretion was higher in the co-culture medium of TLR4-primed cells when compared to the other two groups (data not shown). Jagged 1 and SMAD7 expression within co-cultured cells was measured by flow cytometry (Fig. 9D and E). For the purpose of the analysis, CD45+ cells were considered hPBMCs, and CD90+ adherent cells were considered hMSCs. Jagged 1 expression was elevated in both the hPBMCs and hMSCs populations harvested from TLR4-primed MSC co-cultures when compared to unprimed cultures. SMAD7 expression in both was elevated in TLR3-primed MSC co-cultures when compared to unprimed cultures.

## Discussion

It is now evident that Toll-like receptors (TLRs) are vital in coordinating not only the pro-homeostatic tissue injury responses of immune cells but also that of multipotent mesenchymal stromal cells (MSCs) of various origins. In trying to tease out the molecular details of TLR signaling within human MSCs (hMSCs), we initially observed distinct effects after stimulation of TLR3 when compared with TLR4 activation using our short-term, low-level TLR priming protocol [15]. By use of this protocol in the study presented here, TLR3 stimulation of hMSCs supports their established immunosuppressive effects, while TLR4 activation of hMSCs more consistently provides a pro-inflammatory signature. From these observations, we propose a new paradigm for MSCs that takes its cue from the monocyte literature, that these heterogeneous cells can be induced to polarize into two diverse but homogeneously acting phenotypes [36]. We also contend that many of the conflicting reports on the net effect of TLR stimulation within stem cells can be resolved by taking into consideration the source of cells, their originating species, as well as the time and concentration of TLR agonist exposure. In line with this and the new MSC paradigm, we propose that short-term, low-level exposure with TLR4 agonists polarizes hMSCs toward a pro-inflammatory *MSC1* phenotype important for early injury responses. By contrast, the downstream consequences of TLR3 agonist exposure of hMSCs are its polarization toward an immunosuppressive *MSC2* phenotype essential to later anti-inflammatory responses that help resolve the tissue injury. While our findings that hMSCs can be pro-inflammatory challenge current dogma, a recent report along with the work presented here supports this allegation [47]. Romieu-Mourez *et al.* showed that TLR stimulation in MSCs resulted in the formation of an inflammatory site attracting innate immune cells in neutrophil chemotaxis assays and by the analyses of immune effectors retrieved from TLR-activated MSC microenvironments within mice. We drew our conclusions based on the consistent but different results observed for MSC1 when compared with MSC2 in the many parameters tested and presented. These include dissimilar secretion of cytokines and chemokines (Tables S1 and S2), and differences in differentiation capabilities, ECM deposition, TGFβ signaling pathways, Jagged expression, IDO and PGE_2_ expression, and finally polar opposite effects on T-lymphocyte activation by MSC1 and MSC2.

We provide further support for TLR3 mediated elevated secretion of CCL10 (IP10), CCL5 (RANTES), and IL10, since this effect could be specifically inhibited by dominant-negative TLR3 expression and not TLR4-dominant negative expression (Fig. 1B). However, we found that the enhanced IL6 and IL8 expression after TLR-priming was downstream of both TLR3 and TLR4 activation, and that the secretion of other soluble mediators was indirectly affected by these since no direct effect was noted by the dominant negative strategy (Fig. 1B-note IL4 and data not shown). We add that all the siRNA-driven TLR3 inhibition strategies we attempted were unsuccessful owing to the fact that double stranded RNAs used as the interfering agent are most likely also acting as the agonist for the targeted TLR3 receptor. Inhibition of the expression of TLR3 and TLR4 receptors by nucleofection with knockdown plasmids reduced NF-kB-driven luciferase expression by >90% (data not shown), along with the effect on the soluble mediators. Next, we found that hMSC migration is affected by both the stimulant and the time it is exposed to it (Fig. 2). Whereas TLR-priming promoted hMSC migration, the equivalent short-term exposure with TNFα and CCL5 did not promote migration. Conversely, long-term TLR-priming inhibited hMSC migration but was effective for TNFα and CCL5 mediated migration. We suggest that the short-term, low-level TLR-priming mimics the gradient of danger signals endogenous MSCs encounter and respond to at a distance from the site of injury that draws them to the appropriate target. Once the hMSCs arrive at the site spilling large amounts of these danger signals, migration pathways need to be turned off and the reparitive programs turned on. Transfection of hMSCs with the dominant negative expressing TLR3 and TLR4 plasmids diminished migration by >50% in unstimulated hMSCs as expected. However, poly(I∶C) or LPS stimulation of these transfected cells resulted in further enhancement of migration when compared with unstimulated controls (data not shown). We speculate that specific TLR3 or TLR4 receptor inhibition by the transfected dominant negative expressing plasmids de-represses chemokine or other chemotactic receptors' inhibition downstream from these receptors while potentiating alternative poly(I∶C) or LPS receptors. One potential mechanism that we are currently pursuing to explain this finding is mediated by the suppressors of cytokine signalling (SOCS)1 and SOCS3 within hMSCs. TLR3 stimulation triggers a JAK/STAT signaling cascade indirectly by its induction of type I-interferons resulting in the activation of SOCS 1 and 3. The activation of these proteins downregulate the expression of the chemokine receptor, CXCR4, altering CXCR4-CXCR7-dependent migration of hMSCs. Our study suggests a new role for SOCS, CXCR4, and CXCR7 in hMSC migration (S. Tomchuck unpublished observation).

We hypothesize that polarization of hMSCs by TLR-priming also affects their programming towards tri-lineage differentiation, and that the various reported contrasting effects might also be explained by differences of source, amount, and time of incubation with the TLR-agonists during the induction periods. We measured the effect on hMSC differentiation with our low level TLR agonists maintained for the duration of the induction of hMSC differentiation, and again found evidence that TLR3 and TLR4 have divergent effects on hMSCs. By these methods, we report that TLR3 activation inhibited all of the tri-lineage programs (Fig. 3). TLR4 stimulation of hMSCs inhibited adipogenesis, stimulated osteogenesis, and did not affect chondrogenesis. Others have reported that murine MSCs activation of TLR2 inhibited both differentiation and migration of muMSCs [14]. Liotta *et al.* found no effect of TLR activation on adipogenic, osteogenic or chondrogenic differentiation in hMSCs [21]. Further, in contrast to our study, their report suggested equivalent roles for TLR3 and TLR4 engagement in hMSC immune modulation. Recently, Lombardo *et al.* reported that TLR3 and TLR4 engagement within hADSCs increased osteogenic differentiation but had no effect on their adipogenic differentiation or proliferation [22]. They also report that TLR2, TLR3, and TLR4 ligation does not affect hADSCs ability to suppress lymphocyte activation, in contrast to the Liotta *et al.* report. From these various studies, we argue that specific TLR activation affects many aspects guiding stem cell fates, but unfortunately a consensus on the effect of TLR stimulation and tri-lineage differentiation of stem cells is not possible since some of the experimental details of others' studies were not always included.

Apart from the effects on differentiation, the TLR-priming protocol affected the ability of hMSCs to deposit ECM, another established classical function of these cells. Unlike unprimed hMSCs and TLR4-primed hMSCs that deposited more collagen, TLR3-primed hMSCs deposited more fibronectin (Fig. 4). To help explain these results, we next evaluated TGFβ as an established component of mechanisms that control ECM deposition, and because it is also linked to immune modulation [40], [41], [48]. Indeed, we found that TGFβ, SMAD3, and SMAD7 were affected by TLR-priming of hMSCs (Fig 5 and 6). As expected, enhanced collagen deposition in TLR4-primed hMSCs correlated with TGFβ expression and activation of its downstream signals (phosphoSMAD3). By contrast, TLR3-primed hMSCs that deposited greater levels of fibronectin had decreased TGFβ1 and 3 expression and increased SMAD7 (TGFβ signaling inhibitor) expression. Although we would expect that TGFβ, an immunoregulating factor, would be associated with the TLR3-primed phenotype rather than the pro-inflammatory TLR4-primed one, it is likely that TGFβ plays a smaller role in hMSC immunomodulation than for immune cells. Immune modulatory mechanisms of hMSCs may rely more on local IL10 receptor mechanisms as recently illustrated [24], [49], [50]. Immunomodulation mechanisms controlled by TGFβ appear very complicated, and like TLR-signaling achieve their effects dependent on specific cellular context. For instance, in a recent study, investigators sought to quell inflammation in the brain by manipulation of TGFβ and SMAD3 in immune cells as a new method to prevent Alzheimer's disease. Their strategy surprisingly increased macrophage infiltration in the brain periphery in direct contrast to their original hypothesis, but fortuitously these cells more effectively cleared amyloid plaques [51].

The TGFβ immune dampening effects are also associated with the reprogramming of T-lymphocyte effector cells towards immunosuppressive T-regulatory cells (T-regs). TGFβ cooperates with members of the Notch1 family to regulate the critical transcription factor (Foxp3) to favor Tregs. Additionally, hMSCs are known to recruit and support T-regs as part of their immunedampening effects [52], [53]. TLR3 and TLR4 signaling within MSCs were recently shown to downregulate the Notch1 receptor family member, Jagged 1, and by this method to inhibit T-cell suppression by MSCs [21]. By contrast, we found that by our TLR-priming protocol, Jagged1 expression was elevated in TLR4-primed hMSCs, and dampened only in unprimed or TLR3-primed hMSCs. We speculate that varied concentrations and time of incubations with the TLR-agonists might help explain these differences. Apart from the distinct TLR-driven migration and soluble immune modulators' effects of hMSCs, we observed differences in the expression of IDO and PGE2 secretion (Fig. 8). TLR3-primed hMSCs elaborated elevated levels of both of these when compared with unprimed or TLR4-primed hMSCs. These observations lend further support for our proposed polarization scheme. We are currently evaluating the effect of these mediators in the context of immune responses that TLR-primed hMSCs affect.

We did investigate the immune modulating effect by the TLR-primed cells on T-lymphocyte activation (Fig. 9). In light of the conflicting reports noted above on the effect of TLR3 and TLR4 stimulation on MSCs' ability to suppress T-lymphocyte activation, it was of interest to see what effects our TLR-priming protocol had on this hMSC function. Critical to the main premise of this study, we found that TLR4-primed hMSCs behaved as Liotta *et al*. reported, and inhibited the recognized MSC suppression of T-lymphocyte activation. While in our hands, TLR3-primed hMSCs and unprimed MSCs suppressed T-lymphocyte activation, as expected. Consistent with our proposed new polarization MSC paradigm, TLR4-primed hMSCs (*MSC1*) would support a pro-inflammatory environment including the T-effector cells found in that environment whereas TLR3-primed *MSC2* would favor an immunosuppressive one. In support of this assertion, we have treated murine models of inflammatory lung injury with our *MSC1* and *MSC2* cells, and found by several parameters that, as expected, *MSC1* treatment aggravated the inflammatory injury, whereas *MSC2* improved it, when compared with unprimed hMSC treatments (Dr. Deborah Sullivan unpublished observations). For the T-lymphocyte activation set of experiments, we performed the classical allogeneic co-cultures with direct contact between hMSC-hPBMCs. We did not address the potential of soluble mediators alone in this context. For human-derived MSCs cell-cell contact appears to be essential to their immunomodulatory mechanisms [31], [50]. Indeed, we found contact-dependent secretion by hMSCs of CCL10 (IP-10), CCL5 (RANTES), HGF, and GM-CSF in third party co-cultures with ovarian cancer cell lines and hMSCs (data not shown). We also noted that in the direct cell contact co-cultures performed here, the secretions of these factors followed the same trends, and are consistent with those reported for the hMSCs cultured alone. This finding does not readily explain the contrasting effects by the TLR-primed hMSCs on T-lymphocyte proliferation since we did not measure IL2 levels or other potential T-cell activating factors. More information regarding these effects may be gained from animal disease models where both MSCs and leukocytes (PBMCs) interact and can be more directly tested. Alternatively, a better handle on the molecular details for the important contributions of each TLR-primed cell may be provided in studies using individually marked cell compartments specifically knocked-out for distinct genes.

Interestingly, we noted that in these assays TLR4-primed hMSCs were more readily coated with the round hPBMCs, when compared with unprimed or TLR3-primed hMSCs, in the co-culture assays, after overnight incubation, and throughout the experiment as seen through microscopy. The cell count for this sample group was always greater than that for the other two sample groups (Table 1). We have not investigated the significance of this observation but it goes along with an increase in this sample group of T-cell activation as reported in Fig 9. Our current efforts are focused on determining the specific T-lymphocyte and monocyte effects by the TLR-primed MSCs. Other reports have demonstrated direct cell contact-dependent effects by MSCs to modulate antigen-presenting cells [31], [49], [50]. We aim to extend these observations with the effect of TLR-primed, or polarized, MSC1 and MSC2, on both macrophages and T-lymphocytes. Cell-contact dependent Jagged 1 and SMAD7 expression in the co-cultures correlated with the effects observed for the TLR-primed hMSCs alone (Fig. 7 and 9). The significance of these findings remains to be explored in the studies mentioned above.

Finally, we speculate that until now only an immunosuppressive phenotype has been recognized for current heterogeneous MSC preparations because of the manner in which they are isolated from the host and the way they are expanded in *ex vivo* culture. We reason that the default MSC phenotype must be an immunosuppressive one in order to avoid profound and deleterious consequences from a pro-inflammatory MSC1 phenotype in the context of the hematopoetic stem cells (HSCs) that MSCs maintain and support within the progenitor/stem cell niches both of these cells share. We envision that circulating or quiescent stem/progenitor cells are equipped to respond to environmental cues but must not be actively engaging immune cells or repair cells while circulating throughout the body or maintaining HSCs in the bone marrow niche. In a manner analogous to the immature state maintained for monocytes, dendritic cells, and other immune cells until a response is needed, MSCs are immunosuppressive until a pro-inflammatory role is essential to promote tissue repair. We also surmise that TLR4-priming is not the optimal way to induce the MSC1 phenotype. It is likely that a combination of other factors like interferons or contact with other pro-inflammatory cells and their microenvironments along the lines of that reported by Romieu-Mourez et al. will more readily induce the MSC1 phenotype [47]. Current efforts in the lab are aimed at further defining the MSC1 and MSC2 phenotypes by a comprehensive gene array analyses that will lead to greater clues and more optimal marshalling of the heterogeneous MSC preparations into these two newly defined phenotypes.

In summary, we found that hMSCs polarize into two distinctly acting phenotypes following specific TLR-activation. TLR3-priming specifically leads to enhanced fibronectin deposition, expression of immune dampening mediators, and maintained suppression of T-cell activation. By contrast, TLR4-priming results in collagen deposition, expression of pro-inflammatory mediators, and a reversal of the MSC-established suppressive mechanisms of T-cell activation. Our study challenges current dogma that infused MSCs are only immunosuppressive, and instead suggests that they have more complex immune modulating activity. These findings also provide an explanation for some of the conflicting reports on TLR-activation and its consequence on the immune modulation by stem cells. We also recognize that hMSCs have many cell fates and that this newly described polarizing potential represents an interesting paradigm worthy of further study. In a manner similar to the monocyte field, we caution that although polarization is a convenient way to better define a heterogeneous population of cells that may help in the studying of them, it is not an absolute fate of these cells. We aim to expand the current understanding of MSC biology with these newly defined phenotypes, and to also offer guidance in the improved design of current MSC-based therapies.

## Materials and Methods

### MSCs

Primary human MSCs (hMSCs) were obtained from our collaborators at the Tulane University Center for Gene Therapy. Additionally, hMSCs were obtained from Lonza (Walkersville, MD) to ensure variability of the starting cell population, and to make certain that findings are universal and not unique to single donor pools derived from a unique source, as described [15]. All of the MSC donor preparations from these sources are tested for hematopoetic stem cell markers by the sources and in our lab. All the MSC preparations used in this study are less than 1% positive for CD34 and CD45. MSCs of a passage number no greater than 4 are used in all the experiments to maintain consistency. Also, no less than 5 different unrelated donor MSC pools were tested in all experiments. MSCs from unique donors were tested individually and never pooled with other donors throughout our study.

### TLR Priming Protocol

In this study, LPS (10 ng/mL, Sigma-Aldrich, St. Louis, MO) and poly(I∶C) (1 µg/mL, Sigma-Aldrich) were used as the agonists for TLR4 and TLR3, respectively, as described [15]. Typically, hMSCs are grown to 60–70% confluency in growth medium (DMEM-alpha and 16.5% FCS) prior to the start of an experiment. TLR-agonists are added to fresh growth medium and incubated with the cells for 1 hr. Then the cells are washed twice in growth medium without the TLR-agonists and assayed as described for the experiments. *Please note that we believe based on our observations that short incubation (<1 hr) and minimal TLR agonist exposure at concentrations noted above (or less) are essential to arrive at these phenotypes—which mimic the gradient of danger signals endogenous MSCs encounter and respond to at a distance from the site of injury*.

### LPS Contamination

Rigorous testing for LPS contamination is routinely performed on all the reagents used in this study to avoid spurious conclusions due to this potential TLR-agonist contaminant (Limulus amebocyte lysate chromogenic endpoint assay, Hycult Biotechnologies, The Netherlands). Additionally, all reagents are aliquoted for single or minimal use portions to prevent contamination.

### TLR3 and TLR4 inhibition

hMSCs were grown to 70% confluence, harvested, then transfected with pZERO-hTLR3 and pZERO-hTLR4 (Invivogen), using 250 ng plasmid/1×10^6^ cells (nucleofector). 50 ng pMAX-GFP was transfected alone for control and co-transfected with the pZERO plasmids to monitor transfection efficiency. Each transfection was plated across half of a 24-well plate and allowed to recover for 48 hr. Cells from each transfection were left untreated or stimulated with TLR3 and TLR4 agonists for 1 hr, washed, and incubated for 48 hr. Conditioned medium was harvested and stored at −80°C until analysis. Transfection efficiency was also monitored by co-transfection with 500 ng NF-kB-promoter driven luciferase (LUC)-expressing plasmid (Stratagene/Agilent Technologies LaJolla, CA). Transfection efficiency was determined by these methods to be 30–35% of the cells.

### BioPlex Assays

MSCs were plated at a density of 5×10^4^ in 24-well plates, allowed to adhere overnight, then primed with TLR agonists for 1 hr as indicated. Conditioned medium was collected after 48 hr and analyzed with Bio-Plex Cytokine Assays (Human Group I & II; Bio-Rad, Hercules, CA) following the manufacturer's instructions. These experiments were performed at least three times on three individual MSC donor pools.

### Transwell Migration/Invasion assay

Migration assays were performed in transwell inserts with 8-µm pore membrane filters pre-coated with growth factor-reduced Matrigel™ layer to mimic basement membranes [15], [54]. TLR-primed or unprimed cells were grown to subconfluence (70%) prior to harvesting by trypsinization and labeling with CellTracker™ green (1 µM, Molecular Probes, Eugene, OR) for 1 hr at 37°C. Fluorescently labeled hMSCs (2.5 to 5×10^5^ cells/well in 300 µL) were loaded onto the upper chamber, and 500 µL hMSCs growth medium was loaded onto the bottom chamber. After overnight incubation the upper side of the filters was carefully washed with cold PBS and non-migrating cells remaining were removed with a cotton tip applicator. Fluorescence images of the migrating cells were collected using a Nikon TE300 inverted epifluorescence microscope (DP Controller v1.2.1.108, Olympus Optical Company, LTD; Nikon USA, Lewisville, TX). Each experiment was performed in triplicate with five separate hMSCs donors. Data are expressed as numbers of counted, migrated cells per 200X field micrograph for each sample well, and normalized to those cell counts obtained for the untreated control.

### hMSC Tri-lineage Differentiation Protocols

Modified from [55].

### Chondrogenic Differentiation

hMSCs (2.5×10^5^) were placed into defined chondrogenic medium and gently centrifuged (800×g for 5 minutes) in a 15 mL conical tube where they consolidated into a cell mass or pellet within 24 hours. Chondrogenic medium (CM) consists of high glucose (4.5 g/L) DMEM supplemented with ITS+1 (6.25 µg/mL insulin, 6.2 µg/mL transferrin, 6.25 µg/mL selenous acid, 5.33 µg/mL linoleic acid, 1.25 mg/mL bovine serum albumin), 0.1 µM dexamethasone, 10 ng/mL TGF-β3, 50 µg/mL ascorbate 2-phosphate, 2 mM pyruvate, and antibiotics. TGF- β 3 is prepared fresh from lyophilized powder, and CM in cultures is replaced every third day. At harvest, the samples are fixed in 10% neutral buffered formalin for several hours, and then processed and embedded in paraffin. Sections of chondrogenic pellets were stained with Safranin O to detect the accumulation of proteoglycans.

### Osteogenic Differentiation

hMSCs are cultured at 3×10^4^ cells/well in 6-well plates in growth medium to 70% confluency, then the medium is replaced with medium containing osteogenic supplements (OS). OS consists of 50 µ*M* ascorbate 2-phosphate, 10 m*M* β-glycerol phosphate, and 10^−8^ *M* dexamethasone. After three weeks cells are fixed and stained with 40 m*M* Alizarin Red (pH 4.1) to visualize calcium deposition in the ECM for 10 minutes.

### Adipogenic Differentiation

Adipogenic induction medium (MDI+I medium): 1 µ*M* Dexamethasone and 0.5 m*M* methyl-isobutylxanthine, 10 µg/mL insulin, 100 µ*M* indomethacin, and 10% FBS in DMEM (4.5 g/L glucose) is added to the confluent layer of hMSCs for 48–72 hr. The medium was then changed to adipogenic maintenance medium for 24 hours. Adipogenic maintenance (AM medium) contains 10 µg/mL insulin and 10% FBS in DMEM (4.5 g/L glucose). The cells are treated twice more with MDI+I for a total of three treatments. The cells were washed with PBS and fixed in 10% formalin for 1 h at 4°C, stained for 10–15 minutes at room temperature with a working solution of Oil Red O stain, then rinsed 3x with distilled water.

### Flow cytometry

Human MSCs were harvested and analyzed by flow cytometry with a BD-FACSCalibur flow cytometer (BD Biosciences, San Jose, CA) as described previously [54]. Intracellular antibody staining was achieved after fixation and permeabilization of the cells as indicated by the manufacturer (cytofix/cytoperm buffers, BD Biosciences, San Jose, CA). Isotype controls and untreated or unstained samples were run in parallel, as standard. Analysis of MSC donor pools was performed on a BD-FACSCAlibur (BD Biosciences, San Jose, CA) using BD CellQuest Pro software. Multi-color flow cytometry analysis was performed on a BD LSRII analyzer and analyzed with CellQuest software.

*Primary antibodies:* Isotype-control FITC mouse IgG1K; isotype-control PE mouse IgG1K; isotype-control mouse IgG1K; anti-CD105; -CD166; -CD90; -CD44;-CD34; -CD31; -CD106 (BDBiosciences); -CD45, -CD3, -CD4, - CD8, -CD14, -CD15, -CD19, -CD36, -CD56, -CD123, 235a (eBiosciences), -SMAD3, -phosphoSMAD3, -SMAD7, -JAGGED1 and -JAGGED2 (Cell Signaling Technologies, R&D Biosystems, and Santa Cruz Biotechnologies); β Actin (Sigma-Aldrich, MO, #A-2066).

### Fluorescence Immunocytochemical Analysis (IF)

IF was performed on fixed and permeabilized cells on chamber slides, as before [54]. The primary antibodies were diluted at appropriate concentrations (ratio of 0.5 µg Ab/1×10^6^ cells) and visualized, as standard. Omission of primary antibodies served as negative controls. Micrographs were taken on a Nikon TE300 inverted epifluorescence microscope. Data were presented as stained micrographs and quantified by ImageJ software densitometry analysis from at least three similarly stained sections.

### Transforming growth factor β (TGF)1, 2, and 3 Assays

TGFβ secretion was measured from the conditioned medium by luminex immunoassay as per manufacturer's recommendations (Luminex® Bead immunoassay Kit, LINCOplex from Millipore). The MSCs were pre-treated for 1 hr with LPS or poly(I∶C), washed, and cultured for an additional 48 hr prior to harvesting the spent medium for TGFβ detection.

### Indoleamine 2,3-dioxygenase (IDO) Assay

IDO was measured by real-time PCR analysis of RNA extracted from TLR-primed MSCs incubated for an additional 6 hr prior to RNA harvest. Data are presented by the quantitative comparative CT (threshold value) method, as described before [46].

### HLA-G expression

HLA-G was detected by both western blot analysis and flow cytometry. Western blot with an anti-HLA-G antibody (clone 4H84), and flow cytometry of both membrane and intracytoplasmic molecules were detected with FITC-conjugated Ab directed against anti-HLA-G1/-G5 isoforms (clone MEM-G/9) or HLA-G5 (clone 5A6G7), respectively, as before [33].

### Prostaglandin E2 (PGE_2_) Assay

PGE_2_ was measured from the spent culture medium after 1 hr TLR-agonist priming, wash, and 48 hrs of subsequent culture in growth medium by commercially available ELISA assays (Cayman Chemical, MA).

### Allogeneic mixed lymphocyte and MSC reactions (MLMR)

A variation on published methods was used here to assess alloreactive T-cell proliferation [3], [32]. Human peripheral blood mononuclear cells (PBMCs) were prepared from leucopheresis packs (The Blood Center, New Orleans, LA) by standard centrifugation on a Ficoll Hypaque density gradient. Ten million CFSE-labeled-PBMCs (from at least 5 unrelated donors, Molecular Probes) in the presence or absence of the isolated TLR-primed MSCs or unprimed MSCs were resuspended and stimulated with 1 µg of CD3/CD28 antibody beads (Sigma, St. Louis, MO) at a 10∶1 ratio. After 72 hr, an aliquot was removed for cell counting with trypan blue exclusion as standard and the remainder of the non-adherent cells were then stained with anti-CD8 or CD4 antibody, and the CFSE dilution of the CD8+ cells assessed by flow cytometry analysis (eBiosciences). No less than 100,000 events/sample were collected. Cell surface marker expression of CD4/CD8 was assessed and quantified in arbitrary units as mean fluorescence intensity (MFI) of a live population of cells (propidium iodide negative) labeled with a fluorescence-conjugated monoclonal Ab (eBiosciences).

### Statistical Analysis

Typically, data were represented as average +/− standard error of the mean (S.E.M.). Multiple group comparisons were performed by one-way analysis of variance (ANOVA) followed by the Bonferroni procedure for comparison of means. Comparison between any two groups was analyzed by the two-tailed Student's t-test or two-way ANOVA (Prism4, GraphPad Software Inc. CA). Values of *p*<0.05 were considered statistically significant.

## Supporting Information

Table S1 TLR-regulated gene cDNA arrays. The effect of TLR stimulation on gene expression within hMSCs was analyzed by TLR-pathway focused cDNA array (see “http://www.superarray.com/genetable.php?pcatn=APHS-018A” for details on the arrayed genes). Results are presented as fold changes in gene expression of TLR-primed MSC1 and MSC2 relative to unprimed hMSCs for 6 different donors partially reported in [15].
(0.15 MB RTF)

Table S2 BioPlex Human Cytokine, chemokine and growth factor assays. The hMSCs were pre-treated for 1 hr with TLR agonists (LPS for MSC1 or poly(I∶C) for MSC2), washed and cultured for an additional 48 hr prior to harvesting the spent medium and analysis with Bio-Plex Cytokine Assays following the manufacturer's instructions. Data are expressed in average pg/mL obtained from corrected triplicate measurements with at least 3 MSC donors in four independent experiments. Dominant negative transfected plasmids used were pZero-TLR3 (p0-TLR3) and pZero-TLR4 (p0-TLR4, InvivoGen, San Diego, CA).
(0.08 MB RTF)
