# Supplementary material for: A New Mesenchymal Stem Cell (MSC) Paradigm: Polarization into a Pro-Inflammatory MSC1 or an Immunosuppressive MSC2 Phenotype
Source: PLoS One. 2010 Apr 26;5(4):e10088. doi: 10.1371/journal.pone.0010088 (PMC2859930; doi:10.1371/journal.pone.0010088)
Supplement: Table S1 — TLR-regulated gene cDNA arrays. The effect of TLR stimulation on gene expression within hMSCs was analyzed by TLR-pathway focused cDNA array (see “http://www.superarray.com/genetable.php?pcatn=APHS-018A” for details on the arrayed genes). Results are presented as fold changes in gene expression of TLR-primed MSC1 and MSC2 relative to unprimed hMSCs for 6 different donors partially reported in [15]. (0.15 MB RTF) [file pone.0010088.s001.rtf]

Supplemental Table S1. TLR-regulated gene cDNA arrays.
The effect of TLR stimulation on gene expression within hMSCs was analyzed by TLR-pathway focused cDNA array (see “http://www.superarray.com/genetable.php?pcatn=APHS-018A” for details on the arrayed genes). Results are presented as fold changes in gene expression of TLR-primed MSC1 and MSC2 relative to unprimed hMSCs for 6 different donors partially reported in [15].

Gene	MSC1	MSC2	
			
BTK  	1.461246936	3.294364069	
CASP8 	15.77972327	1	
CCL2 	258.9985122	3191.458118	
CD14 	0.615572207	1	
CD80 	1	1	
CD86 	1	1	
CHUK 	2.045424095	7.889861636	
CLEC4E 	1	1	
CSF2 	22.23560879	1	
CSF3 	1	1	
CXCL10 	29853.24214	1009.902289	
EIF2AK2 	6.717851944	312.9959111	
ELK1 	18.15632106	1	
FADD 	18.86447441	0.946057647	
FOS 	12.04865332	1.071773463	
HMGB1 	8.275233382	1.117287138	
HRAS 	14.71076125	1.892115293	
HSPA1A 	1338.498679	1.802500925	
HSPD1 	19.8903947	0.876605721	
IFNA1 	8.543338824	1	
IFNB1 	806.7623793	37.01402188	
IFNG 	1	1	
IKBKB 	1	0.986232704	
IL10 	14.3005626	1	
IL12A 	6.167988178	1.197478705	
IL1A 	199.8536346	35.26096371	
IL1B 	904.1385139	99.04415959	
IL2 	1	1	
IL6 	829.9036257	20.11221399	
IL8 	5066.592205	69.5510312	
IRAK1 	20.90244787	0.823591017	
IRAK2 	1	12.21007367	
IRF1 	539.3956213	72.50456866	
IRF3 	1	1	
JUN 	21.79008622	1.931872658	
LTA 	15.8718677	1	
CD180 	7.166158732	1	
LY86 	6.636396111	1	
LY96 	45.50647714	0.423372656	
MAP2K3 	26.24553316	0.768437591	
MAP2K4 	14.04125516	0.993092495	
MAP3K1 	27.82674006	0.979420298	
MAP3K7 	12.72152747	1	
MAP3K7IP1 	12.68630476	1.156688184	
MAP4K4 	8.402391453	0.979420298	
MAPK8 	25.54908997	0.939522749	
MAPK8IP3 	9.540070979	1.079228237	
MYD88 	1	4.28709385	
NFKB1 	78.85880809	3.784230587	
NFKB2 	7.729633444	5.897076869	
NFKBIA 	73.15071192	9.253505471	
NFKBIL1 	28.6011252	0.907519155	
NFRKB 	13.93267143	1.189207115	
NR2C2 	11.90256003	1.057018041	
PELI1 	17.83204182	1.591072968	
PPARA 	10.35890784	1.042465761	
PRKRA 	11.05325977	1.035264924	
PTGS2 	121.1289582	2.928171392	
REL 	1	3.182145935	
RELA 	17.20075517	2.173469725	
RIPK2 	83.9582389	28.4429658	
SARM1 	14.4841187	0.460093825	
SIGIRR 	1	1	
SITPEC 	11.94885108	1.094293701	
TBK1 	1	0.907519155	
TICAM2 	1	0.829319546	
TIRAP 	16.70722242	0.598739352	
TLR1 	1	1	
TLR10 	2.984729109	2.203810232	
TLR2 	1	1	
TLR3 	164.1874448	10.77786861	
TLR4 	5.300028068	2	
TLR5 	1	1	
TLR6 	11.03182828	1.515716567	
TLR7 	1	1	
TLR8 	1	1	
TLR9 	20.84457425	1	
TNF 	42.83742928	9.063071082	
TNFRSF1A 	1	1	
TOLLIP 	30.85003027	0.882702996	
TRAF6 	48.7591138	7.06162397	
TICAM1 	42.13070055	3.182145935	

 
