# Supplementary material for: A New Mesenchymal Stem Cell (MSC) Paradigm: Polarization into a Pro-Inflammatory MSC1 or an Immunosuppressive MSC2 Phenotype
Source: PLoS One. 2010 Apr 26;5(4):e10088. doi: 10.1371/journal.pone.0010088 (PMC2859930; doi:10.1371/journal.pone.0010088)
Supplement: Table S2 — BioPlex Human Cytokine, chemokine and growth factor assays. The hMSCs were pre-treated for 1 hr with TLR agonists (LPS for MSC1 or poly(I∶C) for MSC2), washed and cultured for an additional 48 hr prior to harvesting the spent medium and analysis with Bio-Plex Cytokine Assays following the manufacturer's instructions. Data are expressed in average pg/mL obtained from corrected triplicate measurements with at least 3 MSC donors in four independent experiments. Dominant negative transfected plasmids used were pZero-TLR3 (p0-TLR3) and pZero-TLR4 (p0-TLR4, InvivoGen, San Diego, CA). (0.08 MB RTF) [file pone.0010088.s002.rtf]

Supplemental Table S2. BioPlex Human Cytokine, chemokine and growth factor assays. The hMSCs were pre-treated for 1hr with TLR agonists (LPS for MSC1 or poly(I:C) for MSC2), washed and cultured for an additional 48hr prior to harvesting the spent medium and analysis with Bio-Plex Cytokine Assays following the manufacturer's instructions. Data are expressed in average pg/mL obtained from corrected triplicate measurements with at least 3 MSC donors in four independent experiments. Dominant negative transfected plasmids used were pZero-TLR3 (p0-TLR3) and pZero-TLR4 (p0-TLR4, InvivoGen, San Diego, CA).
	unprimed	MSC1	MSC2	unprimed-p0-TL3	unprimed-p0-TL4	MSC1-p0-TL3	MSC2-p0-TL4	MSC2-p0-TL3	MSC2-p0-TL4	
IL1ra	11.1	39.7	120.2							
IL2Ra	0	0	41.3							
IL4	0.5	1.71	3.99	3.99	6.67	3.55	7.79	6.65	8.54	
IL6	414	7287	39987	14,416	9734	15787	9434	22026	13713	
IL8	45	6998	71233	13,055	11432	22533	9837	20345	12994	
IL10	32.8	39.5	33.6	1.96	2.03	0.7	2.8	0.9	2.2	
IL12p40	0	0	11.5							
HGF	256	236	187.9							
IFNg	66.3	336.9	699.4							
CCL10	0	413.3	181777	861	2290	779	2642	1799	24696	
CCL5	15.7	297.4	>35999*	2594	3540	1415	3076	4246	14806	
TNFa	8.1	51.8	501.3	431	339	411	418	460	481	
TNFb	5.5	4.5	3.7							
VEGF	2058	3213.7	2713							

* level for MSC2 was above limit of detection in some of the assays
